# A systematic review on ‘Foveal Crowding’ in visually impaired children and perceptual learning as a method to reduce Crowding

**DOI:** 10.1186/1471-2415-12-27

**Published:** 2012-07-23

**Authors:** Bianca Huurneman, F Nienke Boonstra, Ralf FA Cox, Antonius HN Cillessen, Ger van Rens

**Affiliations:** 1 Bartiméus, Institute for the Visually Impaired, Zeist, the Netherlands; 2 Behavioural Science Institute, Radboud University Nijmegen, Nijmegen, the Netherlands; 3 Department of Developmental Psychology, University of Groningen, Groningen, The Netherlands; 4 Free University Medical Centre, Free University, Amsterdam, the Netherlands

## Abstract

**Background:**

This systematic review gives an overview of foveal crowding (the inability to recognize objects due to surrounding nearby contours in foveal vision) and possible interventions. Foveal crowding can have a major effect on reading rate and deciphering small pieces of information from busy visual scenes. Three specific groups experience more foveal crowding than adults with normal vision (NV): 1) children with NV, 2) visually impaired (VI) children and adults and 3) children with cerebral visual impairment (CVI). The extent and magnitude of foveal crowding as well as interventions aimed at reducing crowding were investigated in this review. The twofold goal of this review is : [A] to compare foveal crowding in children with NV, VI children and adults and CVI children and [B] to compare interventions to reduce crowding.

**Methods:**

Three electronic databases were used to conduct the literature search: PubMed, PsycINFO (Ovid), and Cochrane. Additional studies were identified by contacting experts. Search terms included visual perception, contour interaction, crowding, crowded, and contour interactions.

**Results:**

Children with normal vision show an extent of contour interaction over an area 1.5–3× as large as that seen in adults NV. The magnitude of contour interaction normally ranges between 1–2 lines on an acuity chart and this magnitude is even larger when stimuli are arranged in a circular configuration. Adults with congenital nystagmus (CN) show interaction areas that are 2× larger than those seen adults with NV. The magnitude of the crowding effect is also 2× as large in individuals with CN as in individuals with NV. Finally, children with CVI experience a magnitude of the crowding effect that is 3× the size of that experienced by adults with NV.

**Conclusions:**

The methodological heterogeneity, the diversity in paradigms used to measure crowding, made it impossible to conduct a meta-analysis. This is the first systematic review to compare crowding ratios and it shows that charts with 50% interoptotype spacing were most sensitive to capture crowding effects. The groups that showed the largest crowding effects were individuals with CN, VI adults with central scotomas and children with CVI. Perceptual Learning seems to be a promising technique to reduce excessive foveal crowding effects.

## Background

Visual crowding is a behavioral phenomenon that occurs when identification of an object is seriously undermined by the presence of flankers [1]. Classically, the phenomenon is thought to be caused by contour interaction, attentional factors and/or inaccurate eye movements [2]. The magnitude of the crowding phenomenon or contour interaction in foveal vision (comprising only two degrees of the visual field) can be quantified in two aspects: 1) the maximum distance over which interaction occurs (extent) and 2) the amount of loss in acuity (magnitude) [2]. The disruptive effect of simple surrounds, such as flanking bars, on target recognition is called ‘contour interaction’, and the effect of complex surrounds such as letters is called ‘crowding’ [3].

In normal adult foveal vision, crowding only occurs over very small distances (3–5arcmin[3] or 4–6 arcmin [2]) at the resolution limit and the effect decreases if the target is slightly above the resolution limit (1 arcmin) [2,3]. Other authors mention that crowding effects are absent in foveal vision, but yet already at 2° from the fovea the crowding effect already is quite pronounced [4]. Foveal crowding thus is a controversial term when it is used in the context of adult normal vision [5]. However, extensive crowding effects do occur in the central visual field of strabismic amblyopes [3,5]. Extensive foveal crowding has also been reported in other populations. From literature, we know that contour interaction and foveal crowding are developmental phenomena in individuals with NV and in individuals with abnormal visual input (for example due to central scotomas, visual deprivation during the critical period or fixational instability/nystagmus), but also in individuals with damage of the visual pathways, which is the case in periventricular leukomalacia (PVL) [6]. In visually impaired (VI) children, it could be hypothesized that foveal crowding interferes with the ability to (learn to) read and reading rate and can thus have secondary effects on the acquisition of academic skills. Surprisingly, no interventions have been applied to reduce foveal crowding effects in VI children and adults.

This overview focuses on three groups that show excessive degrees of foveal crowding when compared to adults with NV: (1) children with NV, in this group foveal crowding is present until at least 11 years of age [7], (2) VI children and adults [8-10] and (3) children with a cerebral visual impairment (CVI) [6,11]. In VI individuals, foveal crowding seems to persist much more and much longer than in individuals with NV [10]. The diagnosis CVI is given when 1) there is vision loss in the absence of signs of anterior pathway disease, or 2) when vision loss is greatly exceeding that which could be explained given the findings of ocular examination [12]. We investigated whether Perceptual Learning (PL) is an effective training to reduce crowding effects. PL is based on the notion that practicing visual tasks can lead to dramatic and long-lasting improvements in performing these tasks [13].

This systematic review has a twofold goal: (1) comparing the amount of (foveal) crowding in the three groups of interest, and (2) investigating the potential of PL to reduce crowding effects.

## Methods

### Systematic literature search

Studies were identified by searching electronic databases, scanning reference lists of full text articles that were assessed for eligibility and consultation with experts. The search was applied to PubMed, PsycINFO (Ovid) and Cochrane. The last search was run on 28 May 2012. No limitations regarding year of publication or language were applied. The search was developed by an experienced clinical librarian and the first author of the article. The following search terms were used to search for all databases: visual perception (MeSH term), contour interaction, crowding (MeSH term), crowded, and contour interactions. The search strategy in PubMed is presented in Table 1.

**Table 1:** Search History in PubMed

**Search**	**Most Recent Queries**	**Result**
#10	Search **#3 AND#9**	409
#9	Search **#4 OR #5 OR #6 OR #7 OR #8**	8838
#8	Search **contour interactions[tiab]**	17
#7	Search **crowded[tiab]**	3090
#6	Search **crowding[mesh]**	1792
#5	Search **crowding[tiab]**	5095
#4	Search **contour interaction[tiab]**	40
#3	Search **#1 OR #2**	173402
#2	Search **visual perception[tiab]**	3310

### Study selection

Titles and abstracts were assessed for eligibility by 2 reviewers (BH and FNB), using the inclusion criteria presented in Table 2. All stages of study selection, data extraction, and quality assessment were performed by two independent reviewers (BH and FNB). Disagreements during selection were solved by application of criteria, discussion and consensus. Four articles presenting crowding ratios in children with amblyopia and children with NV were not included. These studies did not focus on our group(s) of interest.

**Table 2:** Inclusion criteria

**Population**	**Children with Normal Vision up to 18 years**
	Children and Adults with Visual Impairment
	Children with Cerebral Visual Impairment up to 18 years
	Adults with amblyopia (addressed for 2 intervention studies)
Intervention	Progress on crowding tasks after Perceptual Learning intervention (n=7)
Type of study	Randomized controlled trials (n=0)
	Non-randomized intervention studies (n=4)
	Cohort studies (n=3)
	Case - control studies (n=4)
	Cross - sectional studies (n=11)
Outcome measurements	Contour interaction area (n=7)
	Crowding ratio (n=8)
	Effects of Perceptual Learning on crowding (n=7)

Seven experimental studies were included which evaluated the effect of an intervention. One study reported an ethics statement and was approved by the University Committee for the Protection of Human Subjects and research was conducted according to the principles expressed in the Declaration of Helsinki. The other six studies reported that subjects gave written informed consent to participate.

### Inclusion criteria

Included quantitative studies focused on: 1) foveal crowding in children with NV up to 18 years, individuals with VI, and children with CVI up to 18 years, or 2) PL studies designed to reduce crowding effects, i.e. reducing contour interaction area or improving crowded acuity (foveal and peripheral). In order to increase data collection about interventions designed to reduce foveal crowding, we also included two intervention studies in adult populations with amblyopia. Studies which included individuals with diagnoses other than those specified above (e.g. dyslexia) were excluded. The term ‘VI individuals’ was used and no age limits were set for this group, because of the scarce amount of studies with regards to VI children.

### Data extraction and quality assessment

Quality of the included studies was evaluated independently by two reviewers (BH and FNB) using criteria for cross sectional and case–control studies [14]. Information for evaluation of the included studies was: number of participants, clear outcome definition, and results (reporting confidence intervals and thresholds in case they were presented).

### Statistical analysis

There were not enough studies using similar paradigms and studies provided too little information on quantitative outcomes to conduct a meta-analysis or sensitivity analysis. Due to methodological heterogeneity, the results of the studies are presented in a narrative way.

## Results

### Results of search and selection process

**Figure 1:**
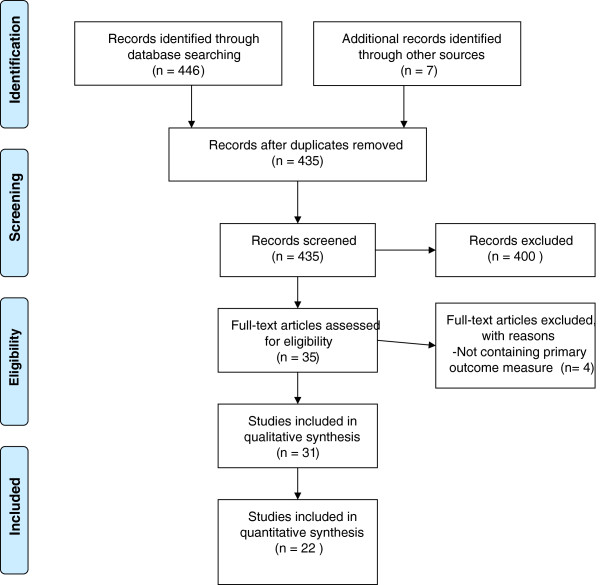
PRISMA 2009 Flow Diagram.

The search of PubMed, PsycINFO (Ovid) and Cochrane databases provided a total of 446 citations. After adjusting for duplicates 435 remained. Seven articles were identified by experts [1,2,15-19]. Of the 435 studies that were identified through database searching, 400 were discarded because they did not meet the criteria (see Table 2). After full text inspection, another 4 articles were excluded because they did not contain our primary outcome measures. Of the included studies, 22 were quantitative studies, 8 additional studies [1-5,12,18,19] were included to clarify the core concepts of (foveal) crowding and contour interaction. See PRISMA flow chart Figure 1. Of the included quantitative studies,4 were non-RCT’s, 3 were cohort studies, 4 were case control studies and 11 were cross-sectional studies.

### Description of included studies

The review focuses on three specific outcome measures: (1) the contour interaction area, (2) the crowding ratio, and (3) effects of PL on crowding. Seven studies were found which measured the contour interaction in the groups of interest. Eight studies were found on crowding ratios. Seven studies were found which measured crowding as an outcome measure after a PL intervention. Table 3 presents the type of observational studies that were included, the characteristics of these studies and the outcome of the studies. Table 4 presents the characteristics of the intervention studies that were included.

**Table 3:** Type of study and outcome for observational studies

**Reference**	**Type of study**	**Number of participants, group (and age).**	**Method**	**Outcome**						
**Fern 1986**^**24**^	Cross-sectional	N = 105 Children NV (2-7y)	Stimulus: Isolated Landolt C/Landolt C with flanking bars at 3 m.	Contour interaction area						
				Children showed the same contour interaction effects as adults at 2.5 × MAR.						
		N = 16 Adults NV								
			Threshold symbol size: 75%							
			Flanker spacing: 2.5 MAR							
			Foveal/Eccentric: foveal							
**Manny 1987**^**21**^	Cross-sectional	N = 13 Children NV (3-4y)	Stimulus: Isolated Landolt C/Landolt C with flanking bars at 3 m.	Contour interaction area (see Figure 2).						
		N = 5 Adults NV								
			Threshold symbol size: 90-95%							
			Flanker spacing: 0–8.52 MAR							
			Foveal/Eccentric: foveal							
**Chung 1995**^**9**^	Case- control	N = 4 Adults CN	Stimulus: Isolated Landolt C/Landolt C with flanking bars at 4.1 m.	Contour interaction area (see Figure 3).						
		N = 6 Adults NV								
			Threshold symbol size: 50%							
			Flanker spacing: 1, 2, 5, or 10 MAR							
			Foveal/eccentric: foveal							
**Pascal 1994**^**8**^	Case- control	N = 6 Adults NV	Stimulus: Isolated Landolt C/Landolt C with flanking bars (3 m or 6 m).	Contour interaction area (see Figure 3).						
		N = 6 Adults idiopathic CN								
			Threshold optotype size: 50%							
		N = 6 Adults with albinism								
			Flanker spacing: 1, 5 MAR							
			Foveal /eccentric: foveal							
**Semenov 2000**^**20**^	Cross- sectional	N = 140 Children NV (3-9y)	Stimulus: Isolated Landolt C/Landolt C with flanking bars at 4.3 m.	Contour interaction area (see Figure 2).						
		N = 4 Adults NV								
			Threshold optotype size: 75%							
			Flanker spacing: 3.75-10 MAR							
			Foveal/eccentric: foveal.							
**Bondarko 2005**^**22**^	Cross- sectional	N = 292 Children NV (8-17y)	Stimulus: Isolated Landolt C, E-letters, Gratings/Landolt C with flanking bars, E-letters with E-letters, Gratings by Gratings at 4.3 m.	Contour interaction						
				The maximum inhibition separation significantly decreased from approximately 2 MAR to 1.5MAR from age 8 to age 17.						
			Threshold optotype size: 75%							
			Flanker spacing: 0–7 MAR.							
			Foveal/eccentric: foveal							
**Jeon 2010**^**7**^	Cross- sectional	N = 59 Children NV (5-, 8-, 11y)	Stimuli: Single Sloan E/Sloan E with gratings at 4.2 m.	Contour interaction area (see Figure 2).						
			Threshold optotype size: 79.1%							
		N = 19 Adults NV								
			Flanker spacing: started at 20MAR (10 reversals)							
			Foveal/eccentric: foveal							
**Atkinson1985**^**26**^	Cross- sectional	Study 1: N = 14 Children NV (5;3–6;2y) N = 9 Mothers Study 2: N = 13 Children NV (3;1–4;1y) N = 8 Mothers	Stimulus: Single Landolt C/Landolt C surrounded by Os and Cs at 1.5-8.3 m.Interoptotype spacing: 50% (line/circular configuration) Foveal/eccentric: foveal	Crowding ratio (see Figure 5).						
**Atkinson 1988**^**27**^	Cross- sectional	N = 47 Children NV (3-4y) N = 12 Adults NVN = 12 Children NV (5-7y)	Stimulus: Single Sheridan Gardener card/5-letter Sheridan Gardener card at 3 m and 6 m.Interoptotype spacing: 50%Foveal/eccentric: foveal	Crowding ratio (see Figure 5).						
**Kothe 1990**^**23**^	Cross-sectional	N = 90 Children NV (4-11y)	Stimulus: Isolated acuity/Regan Repeat Letter acuity/Snellen acuity at 6 m.Interoptotype size: 100%Foveal/eccentric: foveal.	Crowding ratio (see Figure 4).						
**Jacobson 1996**^**6**^	Cross-sectional	N = 13 Children CVI (5-14y)	Stimulus: LH single /LH line at 3 m.Interoptotype spacing = :100%Foveal/eccentric =: foveal	Crowding ratio (see Figure 4).						
**Pike 1994**^**11**^	Cross-sectional	N = 42 Children CVI (2-9y)	Stimulus: Single Sheridan Gardener /7-letter Sheridan Gardener at 6 m.Interoptotype spacing:50%Foveal/eccentric: foveal	Crowding ratio (see Figure 5).						
**Pardhan 1997**^**10**^	Case–control	N = 18VI Adults (42-85y)N = 25 Adults NV (42-85y)	Stimulus: Isolated visual /Regan Repeat Letter Chart, Snellen Line chart at 6 m.Interoptotype spacing: 100% Foveal/eccentric: foveal	Crowding ratio (see Figure 4).						
**Norgett 2011**^**25**^	Cross-sectional	N = 103 Children NV (4-9y)	Stimulus: Single: Kay Picture Single, Single Sheridan Gardiner. 50%: Log MAR crowded acuity, Kay Picture Crowded Log MAR100%: Sonkson Log MAR at 6 m.Interoptotype spacing: see above.Foveal/eccentric: foveal	Crowding ratio (see Figure 4 and 5)						
**Huurneman 2012**^**15**^	Case–control	N = 75 Children NV (4-8y) N = 20VI children without CNN = 38 VI children with CN	Stimulus: C-test, LH-version C-test, LH line test with 25%, 50% and 100% spacing at 40 cm.C-test at 5 m.Interoptotype spacing: see above.Foveal/eccentric: foveal	Crowding ratio						
					C^2.6′^	C (LH)^2.6′^	C^2.6′ far (5m)^	LH^25%^	LH^50%^	LH^100%^
				NV	1.39	1.38	1.22	1.19	1.12	1.12
				VI without CN	1.52	1.56	1.17	1.12	1.12	1.11
				VI with CN	1.76	1.78	1.53	1.25	1.22	1.11

**Table 4:** Type of study and outcome for intervention studies

**Reference**	**Type of study**	**Number of participants, group**	**Method**	**Outcome**
**Chung 2007**^**28**^	Cohort study Perceptual Learning (PL)	N = 8Adults NV	Training: Identifying middle letter trigram at 10° in inferior visual field (0.8× x-height letter separation). 6 sessions = 6000 trials (6 days)Pre-test/Post-test:1) reading speed for 6 print sizes;2) flanked letters identification at 5 separations (0.8×,1×,1.25×,1.6×, and 2×).	1) Maximum reading speed did not improve significantly. Significant reduction in critical print size after training.2) Accuracy for identifying target in a trigram improved significantly (88% improvement).Spatial extent decreased significantly from 1.12x to 0.69x the letter size after training.
**Green 2007**^**29**^	Non-Randomised controlled trial (Non-RCT) Video-game playing (VGP)	Exp. Group: N = 16 Adults NV Control GroupN = 16 Adults NV (all non-videogame players)	Training:-Experimental group: high intensity action videogame;-Control group: less visually intense videogame.30 h training (4–6 weeks) Pre-test/Post-test:Identification middle T trigram at 0°, 10° and 25° (VGPs vs. non-VGPs).	Only the action videogame group showed a significant decrease in crowding region (all eccentricities).No improvement single T acuity after training.
**Huckauf 2007**^**13**^	Non-RCT PL	Training 1: N = 10 (no training); N = 10 (training with feedback target); N = 10 (training with feedback flanker).Training 2: N = 4 Training 3: N = 24 Training 4: N = 11 Adults NV	Training 1: Identify flanked target letters. Always same target/flank combination at 4° and 7°, 1° center-to-center spacing (25 min. training). Training 2: Random assignment trained flankers (letters and unfamiliar symbols). Eccentricities 1°,4° and 7°.2 h/day (3 days; 1980 trials).Training 3: String training at a defined eccentricity of 3° in one of the two visual fields. Participants were measured at three time points: after 144 trials (short training), and an additional 720 trials (long training). Twelve were retested 24 h after training.Training 4: Same as Experiment 3, but also presentation of isolated letters during training and test.	Training 1: Crowding significantly reduces for trained strings and less for untrained strings (specificity effect). No difference between training groups.Training 2: Crowding effects do not reduce when letter combinations differ from trial to trial (specificity effect). Training 3: 16% improvement after short training and 28% improvement after long training. After 24 h, performance was significantly better than at baseline, but did not differ from performance after short or long training. Training 4: Transfer occurred earlier when words were used as stimuli. Isolated letter recognition showed no significant improvement after training, flanked letter recognition improved significantly.
**Maniglia 2011**^**30**^	Cohort study PL	N = 8 Adults NV	Training: Contrast detection of a Gabor target presented in at 4° in the presence of co-oriented and co-aligned high contrast Gabors.160 sessions ≈ 60.000 trials (8 weeks)Pre-test/Post-test1) Visual Acuity2) Crowded acuity3) Contrast sensitivity	1) Visual Acuity did not improve in peripheral vision.2) Crowding reduced significantly in peripheral vision. Observers could better identify a target in a cluttered background.3) Training lateral interactions only reduced contrast sensitivity at the highest spatial frequency used.
**Li 2011**^**17**^	Non-RCT VGP	N = 10(action videogame group) N = 3(non action videogame group) N = 7(crossover control group; 20 h occlusion, 40 h video game therapy) Adults with amblyopia	Training: Action videogame group (n = 10), non-action videogame group (n = 3) and cross-over control group (n = 7).40-80 h videogame playing.Pre-test/Post-test1)Visual Acuity (Bailey-Lovie logMAR charts)2) Positional acuity;3) Spatial attention;4) Stereopsis.	1.1) On average 1.4 to 1.6 lines improvement of acuity after action videogame;1.2) Non-action videogame players improved 1.5 lines on crowded letters and 0.8 lines for single letters. Patching group no improvement in visual acuity after 20 h. Recovery crowded acuity slightly faster than single. Mean crowding index did not significantly improve.2) Positional acuity improved significantly;3) Spatial attention improved significantly;4) Stereopsis improved significantly.
**Sun 2011**^**16**^	Cohort study PL	N = 6Adults NV	Training: Same as Chung^28^.Pre-test/Post-test Identification letter in 2 flanking conditions (unflanked/flanked) crossed with four noise levels.	Accuracy improvement in identifying letters in flanked condition without noise (22%). Training improves efficiency or equivalent input noise in a subject-dependent matter.-Retained improvements after 1–6 months.
**Hussain 2012**^**31**^	Non-RCT PL	N = 10(of which 5 served as a control group that trained after performing 2 pre-tests). Adults with amblyopiaN = 10 (training group)N = 7 (control group) Adults NV	Training: Identifying central target letter (1.4 × threshold size) surrounded by 4 letter in each cardinal orientation. Adults with amblyopia = foveal training. Adults with NV = 4° eccentricity.8–14 sessions (3600–9600 trials)Pre-test/Post-test1) unflanked acuity fellow eye;(2) unflanked acuity amblyopic eye;(3) flanked acuity fellow eye at a spacing of 1.1× letter size; (4) flanked acuities amblyopic eye at spacing of 1.1×, 1.2×, and 1.4× letter size.	1) Unflanked and flanked acuity both significantly improved in the fellow eye. Difference not significant.2) Unflanked acuity improved significantly.3) More progress for flanked than unflanked acuity.Significant improvements on Bailey-Lovie chart on average 1.5 lines.Comparable results for adults with NV in periphery (no improvement for control group). Two follow up participants performed additional sessions and showed a further significant decrease in their crowding ratio’s (after performing 1–11 additional sessions).

### Contour interaction area

**Figure 2:**
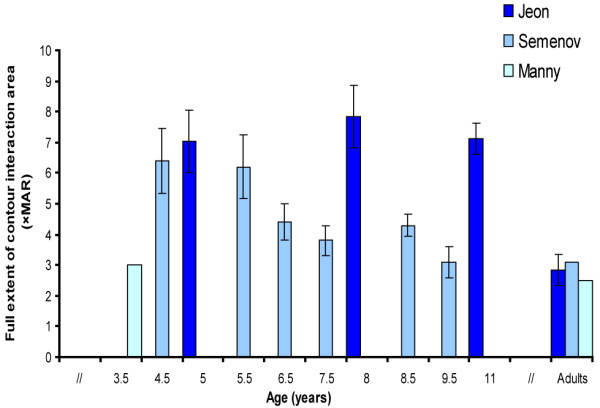
**Full extent of the contour interaction area.** Figure 2 presents the results of three studies that have measured the full contour interaction area in children and adults with NV. Differences between the studies can partially be explained by the different optotypes used. The study by Semenov used Landolt C’s with flanking bars and the study by Jeon et al used E-gratings surrounded by gratings. E-gratings are more difficult to identify than C-rings for children, which might explain the larger contour interaction areas when E-gratings are used [20,23]. Error bars ± 1 s.e.m.

Seven studies on the influence of flanking bars or -contours on object recognition (at the resolution threshold) were found. Five of these were conducted in a population of children with NV [7,20-22] and two were conducted in a population of VI adults [8,9]. Often, the distances over which contour interaction occurs are expressed in steps of the Minimum Angle of Resolution (MAR). Five MAR is equal to the size of one optotype. The outcome of three studies on the full extent of the contour interaction area are presented in Figure 2 [7,20,21].

In three studies on contour interaction in children with NV [7,20,21], the dependent measure was the full extent of the interaction area (the maximum distance over which interaction occurs). Two studies measured the distance at which contour interaction degraded target recognition most [21,22] and one study measured contour interaction at 2.5 MAR [24]. The full extent of the interaction area seemed to be approximately 7 MAR in children (or the size of 1 ½ optotype, inhibition zone size), which is 1.5-3× as large as the interaction area seen in adults) [7,20]. The maximum contour interaction area (distance at which object recognition is most degraded by surrounding contours) was approximately 2.5× MAR according to Bondarko et al.[22] and 0.71 × MAR in the study by Manny et al.[14]. The study by Fern et al. [24] found no difference between contour interaction in children and adults when flankers were placed at 2.5 × MAR.

The three most recent studies showed a clear age effect [7,20,22], with increased contour interaction until adolescence. Two studies found no age effect [21,24]. It should be mentioned that the design of these studies differed with respect to response alternatives. Also, the three most recent studies [7,20,22] were more sensitive at measuring differences than the earlier studies [21,24], because results were based on more trials, had a larger age range, were analyzed per year group and step sizes were smaller. Studies on contour interaction show that the full extent of the contour interaction area is 1.5–3× as large in children with NV as in adults with NV.

**Figure 3:**
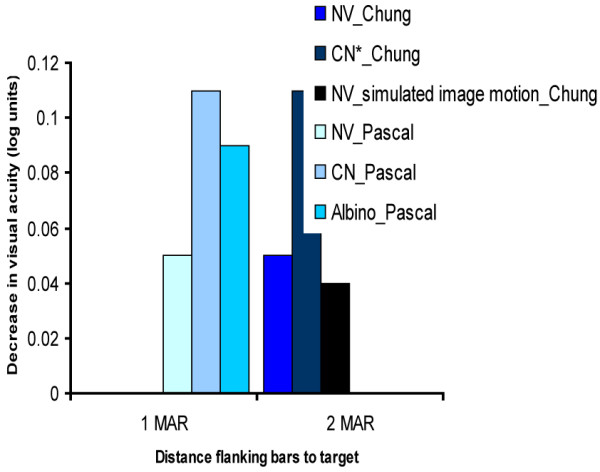
**The magnitude of contour interaction effects at 1 and 2 MAR.** Figure 3 presents the results of two studies which have measured the magnitude of the contour interaction effect in adults with normal vision, adults with congenital nystagmus (CN) and adults with albinism. As can be seen, the magnitude of the effect (defined by the decrease of visual acuity in log units) is the largest in adults with CN in both studies. Standard errors of the mean were not provided.

There were two studies on contour interaction in VI adults [8,9] (see Figure 3). One study compared the full contour interaction area, the point of maximum contour interaction, and the peak magnitude of contour interaction between adults with NV and adults with congenital nystagmus (CN)[9]. Another study focused on the area at which contours caused maximum interaction effects and compared between three subject groups: adults with NV, adults with albinism and adults with CN [8]. Both studies found an increased amount of contour interaction in adults with CN when compared to controls. Adults with albinism did not differ from adults with NV. Adults with CN experience more contour interaction (interaction area is approximately twice as large as in adults with NV). The magnitude of contour interaction in terms of degradation of resolution acuity was also larger in adults with CN (1/2 line in adults with NV and 1.1 line in adults with CN). In the presence of a black background, degradation of resolution acuity was even larger (1.4 line for adults with NV and 2.4 lines for adults with CN). Fixational instability was simulated in adults with NV in a second part of the study [9]. This degraded performance, but did not explain the effect of the contour interaction in individuals with idiopathic CN. The authors mention the possibility of a sensory amblyopia effect as a consequence of the incessant image motion coupled with sizeable astigmatic refractive errors during the period of visual plasticity in early life. Duration of the foveation period, contrast, background colour and orientation played an important role in predicting the amount of contour interaction in the CN group.

### Crowding ratio

**Figure 4:**
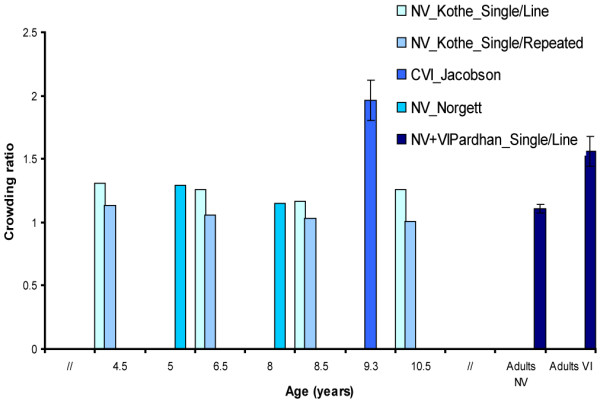
**Crowding ratios measured with charts with 100% interoptotype spacing.** Figure 4 presents the results of four studies which measured crowding ratios in different populations: children and adults with normal vision (NV), children with cerebral visual impairment (CVI), and visually impaired (VI) adults. Children with CVI and adults with VI showed higher crowding ratios than respectively children with NV and adults with NV. Error bars ± 1 s.e.m.

**Figure 5:**
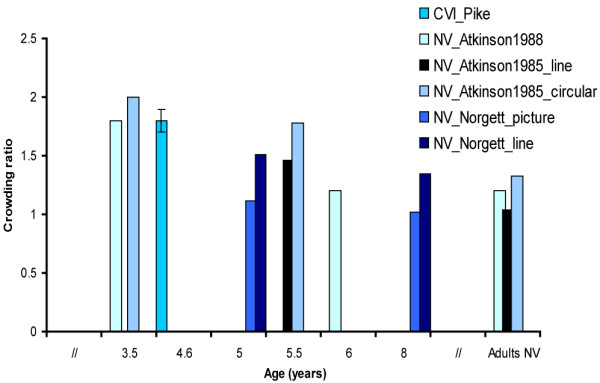
**Crowding ratios measured with charts with 50% interoptotype spacing.** Figure 5 presents the results of four studies which have measured crowding ratios in children and adults with normal vision (NV) and children with cerebral visual impairment (CVI). Line means that the crowding ratio was calculated by dividing the single through the line acuity score and circular means that the crowding ratio was calculated by dividing the single acuity through the acuity score that was measured when a target symbol was surrounded by 6 symbols surrounding the target in all directions. A clear age related reduction of the crowding ratio was observed in children with NV. Error bars ± 1 s.e.m.

Crowding ratios can be calculated by dividing the single decimal line acuity by decimal acuity when optotypes are surrounded. This can be seen as a method to measure the magnitude of the crowding effect. Eight studies were found which measured single and line acuity and crowding ratios were presented or could be calculated from the data presented in the study. As mentioned earlier, due to methodological heterogeneity we could not perform a meta-analysis. However, there were studies using somewhat identical methods. Four comparable studies with interoptotype spacing of 100% are presented in Figure 4 [6,10,23,25] and four studies with interoptotype spacing of 50% are presented in Figure 5 [11,25-27].

Five of these studies were conducted in a population of children with NV [15,23,25-27]. One of these studies compared crowding ratios found in children with NV, VI children without CN and VI children with CN. The study found significantly higher crowding ratios in VI children with CN than in VI children without CN and children with NV [15]. This is the only study we found that measured the crowding ratio for near vision (40 cm) and distance vision (5 m). All other studies only measured crowding for distance vision (1.5–6 m). Another exception is that this study used charts with proportional and charts with absolute interoptotype spacing. The charts with absolute spacing were most sensitive to pick up crowding effects [15]. Another study compared performance on a Repeat letter chart, a Line letter chart and a single letter chart, to investigate whether crowding effects were due to gaze control/selection defects (in which case the Repeat letter chart would show better acuity values than Line acuity charts) or lateral interaction effects (in which case Line chart scores are equal to or better than Repeat chart scores) in children with NV [23]. Children showed higher scores on Repeat letter charts than on Snellen charts and the authors concluded that gaze-selection or gaze-control could be seen as a contributing factor of lower scores on the Snellen chart.

Letter optotypes evoked more crowding than symbols and smaller interoptotype separation resulted in poorer acuity scores (50% vs. 100% interoptotype separation) [25]. The magnitude of the crowding effect, e.g. the influence of crowding on acuity, shows that children with NV score 1–2 lines lower on the visual acuity chart when interoptotype separation is 50% compared to single optotype acuity (depending on age) and the amount of crowding becomes even larger in a circular configuration of target and flankers [26,27]. The large crowding effect at 50% interoptotype separation is in agreement with the studies on contour interaction described above which found maximum interaction effects when bars were placed at 2–2.5× MAR [7,20]. Two studies did not provide the crowding ratio, but presented isolated and line scores, so crowding ratios could be calculated [23,25]. Two other studies did not present standard deviations or standard errors[26,27]. None of these studies presented cut-off scores to indicate extreme crowding, but used group statistics to determine differences [15,23,25-27].

One study compared the crowding ratios of VI adults with those found in age-matched adults with NV [10]. This study compared Repeat Letter acuity with a Line acuity and Single Letter acuity task. In total 83% of VI adults showed visual crowding (defined here as crowding ratio >1). Thirty-nine per cent showed gaze-selection problems and 56% showed lateral interaction effects (see Figure 4 for Single/line ratios). The enhanced crowding effects in this particular population might be due to the use of peripheral fixation, where contour interaction effects are larger. Rehabilitation implications are that if contour interactions are the main cause for a decrease in reading ability, efforts should be directed at designing reading material in such a way that contour interaction effects are minimized. For patients with gaze selection deficits, therapies to improve accurate gaze selection would be beneficial [10].

Two studies have investigated crowding ratios in children with CVI [6,11]. Both studies found enhanced crowding effects in this population. One study [11] investigated patterns of visual impairment in children (n = 42) with different lesions seen on ultrasound before 35 weeks gestational age (severe leukomalacia, large intra ventricular haemorrhages (IVH), or cerebral infarction). Excessive crowding, here defined as a ratio ≥2, occurred in 13 out of 29 children and especially in those with impaired acuity (≤0.30 or ≤6/18) Furthermore, the authors found that visual impairments are more common in association with ischemic lesions (leukomalacia and infarcts) than in association with haemorrhagic lesions, but abnormal crowding ratios were not associated with any particular lesion location on MRI. In contrast, the pattern of visual impairment associated with PVL entails more specific and extensive visual dysfunction [6]. Line acuity for near vision could be tested in 9 of 13 children. A crowding ratio for distance vision could be calculated for 10 children. The crowding ratio was significantly elevated in this group (see Figure 4). Reading was difficult and although the children were able to read short words, they were unable to continue on if the text contained long words on a line. They had difficulties maintaining track, and retracing when they left off. The authors point out that crowding is considered to be one of the major obstacles in fluent reading in children with PVL. Ophthalmological findings report horizontal nystagmus in 12 of 13 children and problems with saccades and pursuit movements.

In sum, it can be concluded that crowding is present in children with NV till adolescent age. The magnitude of the crowding effect, e.g. the influence of crowding on acuity, shows that children with NV score 1–2 lines lower on the visual acuity chart when interoptotype separation is 50% compared to single optotype acuity (depending on age) and the amount of crowding increases in a circular configuration of target and flankers. There seems to be agreement that the following factors are predictive for the extent of crowding in children with NV: gaze selection or gaze control, configuration (circular configuration of stimuli evokes more crowding than linear configuration), maturation of visual areas beyond V1 and cognitive development. In VI adults, acuity is 2 lines lower when optotype separation is 100% compared to single optotype acuity (it was approximately half a line in adults with NV). The effects are due to use of peripheral fixation, gaze selection deficits and lateral interaction effects. In children with CVI, crowding ratios were elevated in both studies (2–3 lines lower score on line acuity chart compared to single acuity with 100% optotype spacing). Specific predictors of the amount of foveal crowding in children with CVI are: kind of lesion (ischemic lesion is associated with poorer visual outcome than hemorrhagic lesions), oculomotor deficits (inability to fixate), presence of nystagmus, and low acuity (≤0.30 or ≤6/18).

### Effects of Perceptual Learning on crowding

Seven articles were specifically about reducing crowding with the help of PL techniques or videogame playing [13,16,17,28-31]. Five of these studies evaluated the influence of PL on the reduction of crowding effects [13,16,28,30,31]. Four studies were conducted in a population of adults with NV [13,16,28,30], and one compared the influence of PL on crowding in adults with amblyopia and adults with NV[31]. We found two studies on videogame playing and the reduction of crowding [16,29]. One was conducted in a population of adults with NV [29] and one was conducted in a population of adults with amblyopia [16].

A non-Randomized Controlled Trial (non-RCT) investigated the effect of PL on the reduction of crowding [13]. In this PL study [13], the training period was very short (25 minutes), the groups were relatively small (N = 10) and the authors did not measure effects of PL on improvements on acuity measures. However, there was improvement on flanked letter recognition. A specific learning effect for trained strings was found. A second non-RCT showed that foveal crowding ratios and visual acuity in adults with amblyopia and peripheral crowding ratios in adults with NV improved significantly after 8–14 sessions of PL (1.5 lines on average) [31]. Three cohort studies on PL and the reduction of crowding effects in the periphery showed that, in adults with NV, accuracy for identifying flanked letters improved significantly [16,28,30], and isolated letter acuity did not improve [30], and the reduction in crowding effects was retained up to at least 6 months [16,28]. Again, sample sizes were very small in this study (N = 6–8). Thus, there are indications that PL reduces crowding effects, but it also has the potential to improve flanked ánd unflanked acuity after training on a crowded letter identification task in amblyopic foveal and normal peripheral vision [31].

A non-RCT was conducted in a population of adults with NV and evaluated whether (action) videogame playing (VGP) has the potential to reduce crowding effects in central and peripheral vision [29]. This study found that crowding effects decreased significantly after action VGP, but crowding effects did not decrease in the control group which trained with a less visually-intense non action videogame. However, the number of participants was relatively small (N = 16), and the effect size of the reduction of crowding was rather small (η_*p*_^2^ = .14). Isolated acuity did not improve after VGP. A second non-RCT study, with a more extensive training period conducted in a population of adults with amblyopia, showed significant improvement in flanked ánd unflanked visual acuity after 40–80 h of (action) videogame playing (on average 1.5 letter lines) [17]. There was no difference in the amount of improvement in flanked and unflanked acuity. The mean crowding index did not improve significantly after videogame playing [17], as was seen in the PL study in adults with amblyopia [31]. The improvement in visual acuity was found for action videogames and non-action videogames. Although this study showed impressive recovery in visual acuity that is about 5-fold faster than that expected after occlusion therapy, the authors also point out that the study contains several limitations: small sample size, lack of randomization, and differences in number of groups. The conclusion is that a large-scale randomized study is needed to confirm the therapeutic value of videogame treatment in clinical situations.

There is stronger evidence for PL as an effective method to specifically reduce crowding effects than VGP. Although it has never been studied, it is plausible that PL could improve visual functioning in children with a (cerebral) visual impairment, because the factors that seem account for foveal crowding in this group are: fixational instability, gaze selection problems, poor contrast sensitivity, poor visual acuity, large interaction areas (possibly due to amblyopia effects) and short foveation periods. The above studies illustrated the prospects of PL on: reducing critical spacing (or contour interaction areas) or improvement of recognition for crowded stimuli [13,16,17,28-31], improvement on clinical measures of visual acuity [17,31], improving contrast sensitivity [30], improving ocular alignment and training non retinotopic higher brain processes engaged in attention and decision making [17].

## Discussion

The goal of the present review was to compare studies which measured foveal crowding in three specific groups and explore possible interventions for crowding in children with a (cerebral) visual impairment. An important and striking conclusion must be that no interventions have been evaluated in our groups of interest, despite the abnormal crowding ratios in children with a (cerebral) visual impairment [9,11,15]. It is also surprising that there are so few quantitative studies which have measured crowding in the VI child population and studies use different cut-off points to determine what quantifies abnormal crowding.

The first goal of this overview was to describe the manifestation of the crowding phenomenon in children with NV (1), the VI group (2) and children with CVI (3), because it is conceivable that different factors and mechanisms are involved in these groups. However, different paradigms were used to measure crowding (methodological heterogeneity) and therefore results were presented in a narrative way. Factors that were identified to influence crowding in children with NV are: development of gaze selection/control [15], configuration of the stimulus [26], cognitive development [26,27] and maturation of cortical structures beyond VI that are involved in the integration of local information [7]. Factors influencing crowding in the VI group were: fixational stability [7,9], background color [9], contrast [8], orientation [8], and the presence of central scotomas [10]. In the VI group, there is consistent evidence that individuals with CN experience contour interaction over larger interaction areas and performance is more degraded by nearby contours in this group than in a control group with NV[9]. There is one study which shows that adults with a visual impairment show elevated crowding ratios, this study mentions that these results could are due to eccentric fixation in this group[10]. There is one study which measured crowding in VI children, and this study found significantly higher crowding ratios for VI children with nystagmus than VI children without nystagmus and children with NV [15]. When interoptotype spacing is small, children with NV show a smaller loss of acuity than VI children. It might be reasoned that children with a congenital visual impairment may have developed amblyopia as a secondary symptom to their altered visual development [8,9]. Findings in the CN group suggest that this group could directly benefit from reduced contrast, a white background and proportionally larger interoptotype spacing [9]. Only one study could be found on crowding in the presence of albinism and this study provided no evidence of increased crowding compared to controls [8]. Children with CVI, especially those with PVL, experienced abnormal crowding effects which can be related to the degree of and kind of cortical trauma (ischemic lesions and infarcts seem to be more predictive of abnormal visual function than hemorrhages) and ability to fixate [6,11]. This is a consistent finding in the studies that were included for this overview. Visual functioning in children with CVI is affected in different areas: visual fields are constricted (due to damage in the optic radiation), the majority of children exhibit nystagmus or strabismus, subnormal visual acuity, excessive crowding, and problems in simultaneous perception [6,11].

The last section was about interventions that have been designed to reduce crowding. Seven studies were found that specifically aimed at reducing crowding [13,16,17,28-31]. The intervention studies that were found have a small sample size [13,16,17,28-31], and we found no interventions for our groups of interest. The interventions discussed above therefore should be seen as pilot studies. The small sample size and the differences in group numbers might bias the outcome and this review emphasizes the need for large randomized controlled studies. However, the studies that we did found showed that the PL techniques was more effective in specifically reducing crowding effects than the videogame playing studies. Three studies demonstrated that foveal resolution in adults with NV and adults with amblyopia can be enhanced by training [17,29,31]. The technique could be applied to reduce foveal crowding effects in individuals with congenital nystagmus, central scotomas, and children with CVI. Crowding effects, or inappropriately large integration areas, in the normal periphery and foveal amblyopic vision have been explained by extended pooling at a stage following the stage of feature detection [18,19]. This review illustrates that there is accumulating evidence that the normal periphery and foveal amblyopic vision can be fine-tuned by excessive presentation of challenging (crowded) stimuli. Because of sensory amblyopia effects [9] and fixational instability in our groups of interest [6,10,11,15], PL could also work for children with a (cerebral) visual impairment. This review illustrates that there is a need for RCT’s to investigate the value of PL in populations that experience excessive crowding effects (VI individuals with secondary amblyopia effect or nystagmus, children with CVI).

Thus, foveal crowding seems to be associated with an underdeveloped and/or understimulated visual system and practicing those areas of impairment can possibly produce improvements. Higher and lower level visual functions are interdependent and work together. Weaker lower level functioning in VI individuals, may lead to higher level impairments like the secondary amblyopia effect [8,9]. We have seen that gaze control and fixational stability play an important role in the amount of crowding in children with nystagmus and children with CVI. This fixational instability does not tell us the whole story. Research has delivered evidence that more contour interaction is present when contrast is stronger [8]. Whether foveal crowding can be reduced by practicing challenging tasks (such as letter identification in a busy visual field) and improving oculomotor control (through special designed games) is an interesting and novel question. PL literature on crowding stresses the importance of looking at individual capacities and when there are specific areas of impairment, these are the areas that the training should focus on.

## Conclusions

This overview shows that there is still much to learn about foveal crowding in children with a (cerebral) visual impairment and it is hard to compare findings because paradigms are different in nature. There seem to be differential mechanisms at play in the different subtypes of visual impairments. Evidence was found for enhanced crowding effects in individuals with CN, VI adults with central scotomas and children with CVI. Although literature was scarce, children with CVI showed the highest crowding ratios. Oculomotor control seems to play a crucial factor in predicting the amount of crowding. Interventions should be designed with these mechanisms kept in mind. Although there is a lack of large-scale randomized controlled trials on PL in patient populations, the findings presented in this review indicate that Perceptual Learning is an effective technique to reduce peripheral crowding in adults with NV and foveal crowding in adults with amblyopia.

## Competing interests

The authors declare that there is no competing of interest.

## Authors’ contributions

Literature screening and selection was performed by BH and FNB. Data extraction and synthesis was performed by BH and FNB. Preparation of the first draft of the manuscript was done by BH and review and approval of the manuscript was performed by NB, AC, GR and RC. All authors read and approved the final manuscript.

## Pre-publication history

The pre-publication history for this paper can be accessed here:

http://www.biomedcentral.com/1471-2415/12/27/prepub
